# Preventive Effects of Collagen-Derived Dipeptide Prolyl-Hydroxyproline against Dexamethasone-Induced Muscle Atrophy in Mouse C2C12 Skeletal Myotubes

**DOI:** 10.3390/biom13111617

**Published:** 2023-11-05

**Authors:** Yoshifumi Kimira, Konosuke Osawa, Yoshihiro Osawa, Hiroshi Mano

**Affiliations:** Department of Clinical Dietetics and Human Nutrition, Faculty of Pharmacy and Pharmaceutical Sciences, Josai University, 1-1 Keyakidai, Sakado-shi 350-0295, Japan

**Keywords:** collagen-derived peptide, Prolyl-hydroxyproline, muscle atrophy, mouse C2C12 skeletal myotubes, ubiquitin ligases

## Abstract

Glucocorticoids, commonly used to manage inflammatory diseases, can induce muscle atrophy by accelerating the breakdown of muscle proteins. This research delves into the influence of Prolyl-hydroxyproline (Pro-Hyp), a collagen-derived peptide, on muscle atrophy induced with dexamethasone (DEX), a synthetic glucocorticoid, in mouse C2C12 skeletal myotubes. Exposure to DEX (10 μM) for 6 days resulted in a decrease in myotube diameter, along with elevated mRNA and protein levels of two muscle-atrophy-related ubiquitin ligases, muscle atrophy F-box (MAFbx, also known as atrogin-1) and muscle ring finger 1 (MuRF-1). Remarkably, treatment with 0.1 mM of Pro-Hyp mitigated the reduction in myotube thickness caused by DEX, while promoting the phosphorylation of Akt, mammalian target of rapamycin (mTOR), and forkhead box O3a (Foxo3a). This led to the inhibition of the upregulation of the ubiquitin ligases atrogin-1 and MuRF-1. These findings indicate the potential significance of Pro-Hyp as a promising therapeutic target for countering DEX-induced muscle atrophy.

## 1. Introduction

Skeletal muscle atrophy is defined as a reduction in both the size and mass of muscle tissue, which occurs when protein breakdown surpasses protein synthesis [1]. This process leads to exercise intolerance, impeding daily activities due to muscle weakness and fatigue, ultimately compromising the overall quality of life [2].

Synthetic glucocorticoids have demonstrated utility in managing a wide range of inflammatory conditions. Dexamethasone (DEX), a synthetic glucocorticoid, has been used as a therapeutic intervention for various conditions due to its potent anti-inflammatory and protective properties against autoimmune diseases [3]. Nevertheless, despite these benefits, administering high doses and prolonged usage of DEX can result in severe side effects, including the development of muscle atrophy [4]. DEX acts as a catabolic regulator of skeletal muscle by upregulating the transcription of two muscle-specific ubiquitin E3 ligases, muscle atrophy F-box (MAFbx, also known as atrogin-1) and muscle ring finger 1 (MuRF-1), thereby contributing to the increased proteolysis seen during muscle atrophy [5]. Previous studies have indicated the involvement of forkhead box O (Foxo) transcription factors in the regulation of atrogin-1 and MuRF-1 expression during DEX-induced muscle atrophy [6]. Moreover, DEX induces muscle atrophy in mouse myocytes by inhibiting the phosphorylation of muscle protein synthesis factors, including Akt and the mammalian target of rapamycin (mTOR) [7].

Collagen is a pivotal extracellular matrix protein predominantly found in dense connective tissues such as skin, bone, tendons, or fascia [8,9,10]. Collagen turnover gives rise to bioactive collagen peptides (CPs) through enzymatic degradation [11,12]. Collagen features at least one common Glycine (Gly)-X-Y repeat domain, where X and Y are predominantly Proline (Pro) and hydroxyproline (Hyp), respectively. A multitude of bioactive peptides derived from collagen include Hyp in their sequences [13,14]. Certain functional peptides within CPs are absorbed into the bloodstream as oligopeptides [14,15], exerting diverse bioactive effects. Numerous studies have documented the favorable impacts of orally administered CPs on osteoporosis, osteoarthritis, and knee joint discomfort [16,17,18]. One notable collagen-derived bioactive peptide, Prolyl-hydroxyproline (Pro-Hyp), has been demonstrated to enhance the migration of fibroblasts from mouse skin and facilitate the differentiation of osteoblasts, tendon cells, and chondrocytes [19,20,21,22,23]. Another significant collagen-derived bioactive peptide, hydroxyprolylglycine (Hyp-Gly), has been found to induce muscle hypertrophic effects in C2C12 cells [24].

In addition to its diverse bioactive effects, CP has been recently reported to affect muscle mass positively. In a randomized controlled trial involving premenopausal women (ages 18–50 years), CP supplementation along with resistance training for 12 weeks significantly increased fat-free mass and hand-grip strength [25]. Furthermore, CP supplements also led to increased fat-free mass in middle-aged, untrained men (ages 30–60 years) undergoing resistance training [26].

CP supplementation has also demonstrated an impact on muscle breakdown. Previous research indicated that the intake of 15 g of specific collagen peptides notably increased fat-free mass and leg muscle strength following resistance training in older men with sarcopenia [27]. Recent animal experiments further indicated that CP administration along with treadmill exercise effectively prevented DEX-induced muscle atrophy in mice [28], and CP supplementation also improved age-related sarcopenia in middle-aged mice [29].

Hence, CP may have a significant role in mitigating muscle atrophy. Nonetheless, the influence of specific sequence peptides within CP on suppressing muscle atrophy remains unknown. In this study, we investigated the preventive effects of Pro-Hyp on DEX-induced muscle atrophy in C2C12 myotubular cells.

## 2. Materials and Methods

### 2.1. Cell Culture and Treatment

The C2C12 myoblast cell line mouse was obtained from the RIKEN Cell Bank (Tsukuba, Japan). C2C12 myoblast cells were cultured in Dulbecco’s Modified Eagle Medium (DMEM, Cat. #11885-084, Gibco, Thermo Fisher Scientific, Waltham, MA, USA) containing 10% fetal bovine serum (FBS, Cat. #7524, Nichirei Biosciences, Tokyo, Japan) and 100 U/mL of penicillin, and maintained in a humidified incubator at 37 °C under a 5% CO_2_ atmosphere until they reached 80–90% confluence. Subsequently, to induce differentiation into myotubes, the medium was exchanged for DMEM containing 2% FBS for 6 days, with medium changes every 2 days. For an immunofluorescence analysis, fully differentiated C2C12 cells were treated with 10 μM of DEX (Cat. #D1756, Sigma-Aldrich, St. Louis, MO, USA) for 6 days to induce muscle atrophy. Pro-Hyp (0.01, 0.1 mM, Cat. #4001630, Bachem, Bubendorf, Switzerland) was diluted in the DMEM medium and co-treated with DEX for 6 days. For qRT-PCR and Western blot analyses, fully differentiated C2C12 cells were treated with DEX (10 μM) and Pro-Hyp (0.01, 0.1 mM) for 24 h. DEX was dissolved in dimethyl sulfoxide (DMSO, Cat. #D8418, Sigma-Aldrich, St. Louis, MO, USA), and DMSO (0.01%) was used as the vehicle for DEX. The dose of Pro-Hyp was selected based on the fact that the concentrations used in previous reports of cellular experiments on the physiological effects of Pro-Hyp have also involved 0.1 mM as the upper limit [30].

### 2.2. Immunofluorescence Analysis

C2C12 myotubes were fixed in 4% paraformaldehyde in phosphate-buffered saline (PBS, Cat. #D163-20145, Wako Pure Chemical Industries, Ltd., Osaka, Japan) for 10 min at room temperature and washed twice with 0.1% Triton X-100 (Cat #160-24751, FUJIFILM Wako Pure Chemical, Osaka, Japan) in PBS. They were subsequently permeabilized with 0.1% Triton X-100 in PBS for 20 min. At room temperature, the cells were blocked with 3% bovine serum albumin in PBS for 1 h. Then, the cells were incubated with the myosin heavy chain (MHC) primary antibody (1:400, Sigma-Aldrich, St. Louis, MO, USA) overnight at 4 °C. After washing three times with 0.1% Tween 20 (Cat #167-11515, FUJIFILM Wako Pure Chemical, Osaka, Japan) in PBS (PBST), the cells were incubated with a secondary antibody conjugated to fluorescein isothiocyanate (FITC, 1:100, Cat. #sc2099, Santa Cruz, TX, USA) for 1 h at room temperature. After washing three times with 0.1% PBST, the nuclei were counterstained with 4′,6-diamidino-2-phenylindole (DAPI, Cat. #S36964, Thermo Fisher Scientific, MA, USA). Fluorescent images were analyzed using a BZ-810 fluorescence microscope (Keyence, Osaka, Japan). Fifty myotubes/group were measured randomly from five different fields. The thickest portion of each myotube was chosen for diameter measurement using BZ-H4M image analysis software (version 1.4.1.1, Keyence, Osaka, Japan).

### 2.3. RNA Preparation and Quantitative RT-PCR (qPCR)

Total RNA was extracted from the cells using the RNeasy Mini Kit (Qiagen, Hilden, Germany), and cDNA was synthesized from 5 mg of mRNA using the Prime Script Reagent Kit (Takara Bio Japan, Otsu, Japan). qPCR was performed with TB Green^®^ Fast qPCR Mix (Takara Bio Japan, Otsu, Japan). Glyceraldehyde 3-phosphate dehydrogenase (GAPDH) was used as the internal control for normalizing the expression of target genes. The quantitative PCR primers were as follows: 5′-TGTCTGGAGGTCGTTTCCG-3′ (forward) and 5′-CTCGTCTTCGTGTTCCTTGC-3′ (reverse) for MuRF-1; 5′-GAGTGGCATCGCCCAAAAGA-3′ (forward) and 5′-TCTGGAGAAGTTCCCGTATAAGT-3′ (reverse) for atrogin-1; 5′-AGTGAATGAGGCCTTCGAGA-3′ (forward) and 5′-GCA TCTGAGTCGCCACTGTA-3′ (reverse) for MyoD; 5′-ACTCCCTTACGTCCATCGTG-3′ (forward) and 5′-CAGGACAGCCCCACTTAAAA-3′ (reverse) for Myogenin; 5′-ACTGAGCAAGAGAGGCCCTA-3′ (forward) and 5′-TGTGGGTGCAGCGAACTTTA-3′ (reverse) for GAPDH.

### 2.4. Western Blot Assay

Cells were washed twice with ice-cold PBS and then lysed with a radioimmunoprecipitation assay (RIPA) buffer consisting of 25 mM Tris-HCl (pH 7.6), 150 mM NaCl, 1% NP-40, 1% sodium deoxycholate, and 0.1% sodium dodecyl sulfate (SDS), and containing a protease inhibitor cocktail (Cat. # 87786, Thermo Fisher Scientific, Waltham, MA, USA). Cell lysates were centrifuged at 15,000 rpm for 20 min, and the supernatants were collected as protein samples. The protein concentration of each sample was measured with the bicinchoninic acid (BCA) Protein Assay Reagent (Thermo Fisher Scientific, Waltham, MA, USA). Proteins were separated with sodium dodecyl-sulfate polyacrylamide gel electrophoresis (SDS-PAGE) and transferred to polyvinylidene difluoride (PVDF) membranes using the Trans-Blot Turbo transfer system (Bio-Rad, Hercules, CA, USA). After blocking with 5% skim milk in TBS-T consisting of 10 mM Tris-HCl (pH 7.4), 1.37 M NaCl, and 0.1% Tween 20 for 30 min at room temperature, the membranes were incubated with rabbit anti-atrogin-1 (Cat. #ab168372, abcam, Cambridge, UK), rabbit anti-MuRF-1 (Cat. #ab172479, abcam, Cambridge, UK), rabbit anti-ubiquitin (Cat. #3936; Cell Signaling Technology, Danvers, MA, USA), rabbit anti-Akt (Cat. #2920; Cell Signaling Technology, Danvers, MA, USA), rabbit anti-phospho-Akt (Cat. #4060; Cell Signaling Technology, Danvers, MA, USA), rabbit anti-mTOR (Cat. #2983; Cell Signaling Technology, Danvers, MA, USA), rabbit anti-phospho-mTOR (Cat. #2971; Cell Signaling Technology, Danvers, MA, USA), rabbit anti-Foxo3a (Cat. #12829; Cell Signaling Technology, Danvers, MA, USA), rabbit anti-phospho-Foxo3a (Cat. #9496; Cell Signaling Technology, Danvers, MA, USA), or rabbit anti-β-actin (Cat. #4970; Cell Signaling Technology, Danvers, MA, USA) 1/1000 diluted in a blocking buffer for 1 h at room temperature. The membranes were washed with TBS-T and then incubated for 45 min at room temperature with HRP-conjugated rabbit anti-mouse IgG (Cat. #7074; Cell Signaling Technology, Danvers, MA, USA) 1/2000 diluted in TBS-T. To reprobe with another antibody, blots were incubated in a stripping buffer consisting of 62.5 mM Tris-HCl (pH 6.7), 2% SDS, and 100 mM 2-mercaptoethanol at 50 °C for 30 min and analyzed as described above. Labeled proteins were detected with EZ West Lumi plus (ATTO, Tokyo, Japan). Band intensities were determined using ImageJ software (version 1.54 g, National Institutes of Health, Bethesda, MD, USA).

### 2.5. Statistical Analysis

Data are expressed as means ± standard deviation (SD). Statistical analyses were performed using SPSS Statistics for Mac, Version 25.0 (IBM Corp., Armonk, NY, USA). Differences among multiple groups were compared using one-way analyses of variance (ANOVA) with Tukey post hoc tests. Values with *p* < 0.05 were considered statistically significant.

## 3. Results

### 3.1. Pro-Hyp Suppressed DEX-Induced C2C12 Myotube Atrophy

**Figure 1:**
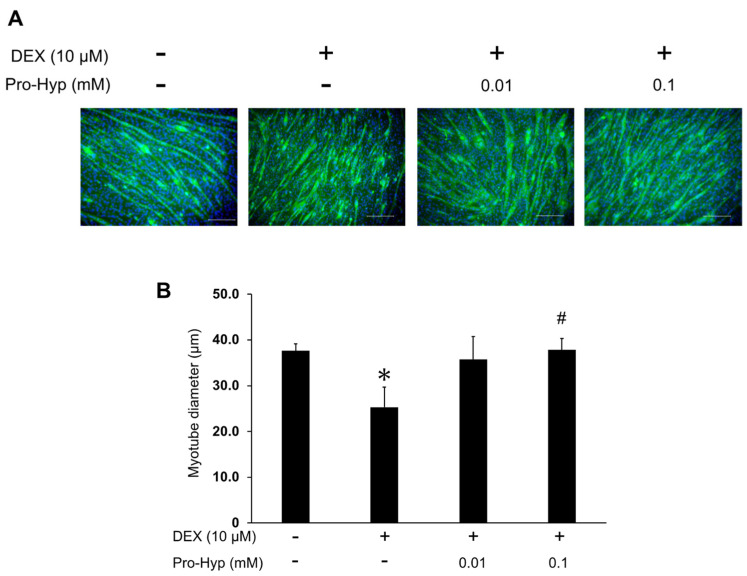
Effects of Pro-Hyp on myotube diameter in DEX-stimulated C2C12 myotubes. (**A**) Representative images of C2C12 myotubes treated with 10 μM of DEX and Pro-Hyp (0.01 mM and 0.1 mM). Fixed cells were reacted with an anti-MHC antibody and a fluorescence-labeled secondary antibody (green). The nuclei were stained with DAPI (blue). The scale bar represents 250 μm. (**B**) Comparison of myotube diameters among the four treatment groups. Data are expressed as means ± SD. * *p* < 0.05 vs. non-treated controls, # *p* < 0.05 vs. DEX-treated groups. *p* < 0.05.

To evaluate the protective effect of Pro-Hyp on muscle atrophy, the myotube diameters were analyzed with immunofluorescence staining. Figure 1A displays representative photographs of the treated myotubes. Compared to the control group, myotube diameter was reduced by 23.4% after 6 days of treatment with 10 μM of DEX, confirming that DEX effectively induced muscle atrophy. However, in the DEX + 0.01 mM Pro-Hyp treated group, the myotube diameter recovered to 96.3% of the control value. Furthermore, myotube diameter in the DEX + 0.1 mM Pro-Hyp group showed a significant increase compared to the DEX group and was similar to the control group (Figure 1B). These results indicate that Pro-Hyp had protection activity against muscle atrophy in DEX-treated C2C12 myotubes.

### 3.2. Pro-Hyp Ameliorates Muscle-Atrophy-Associated Genes in DEX-Induced Myotube Atrophy

**Figure 2:**
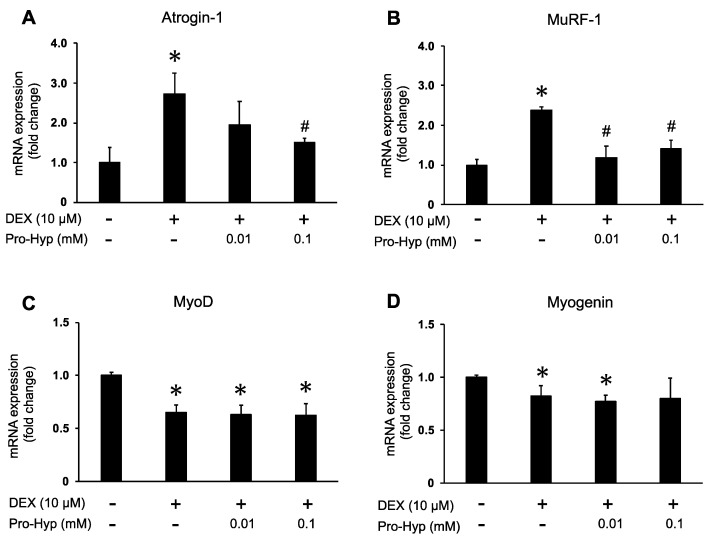
Effects of Pro-Hyp on muscle-atrophy-associated genes and myotube-differentiation-related genes in DEX-stimulated C2C12 myotubes. C2C12 myotubes were treated with 10 µM of DEX and Pro-Hyp (0.01 mM and 0.1 mM) for 24 h. (**A**) Atrogin-1, (**B**) MuRF-1, (**C**) MyoD, and (**D**) Myogenin mRNA levels were examined with qPCR. GAPDH was used as an internal control. Data are expressed as means ± SD. * *p* < 0.05 vs. non-treated controls, # *p* < 0.05 vs. DEX-treated groups. *p* < 0.05.

To investigate the effect of Pro-Hyp on muscle atrophy in DEX-treated C2C12 myotubes, the mRNA expression levels of atrogin-1 and MuRF-1, muscle-atrophy-related ubiquitin ligases, were assessed through qPCR. Upon exposure to a medium containing DEX, mRNA levels of atrogin-1 and MuRF-1 were significantly higher compared to the control group (Figure 2A,B). However, the DEX + 0.01 mM Pro-Hyp treated group resulted in a 20% reduction in atrogin-1 mRNA expression compared to the DEX group. The DEX + 0.1 mM Pro-Hyp treated group significantly reduced the mRNA expression of atrogin-1 compared to the DEX-treated C2C12 myotubes. Furthermore, Pro-Hyp (0.01 and 0.1 mM) treatment significantly reduced the expression of MuRF-1 compared to the DEX-treated C2C12 myotubes. These findings indicate that Pro-Hyp is associated with downregulating muscle-atrophy-related ubiquitin ligases. We also assessed the mRNA expression levels of MyoD and Myogenin, which are myogenic markers, and observed that exposure to a medium containing DEX significantly decreased the mRNA levels of MyoD and Myogenin in comparison to the control group (Figure 2C,D). However, the simultaneous addition of DEX and Pro-Hyp did not affect the DEX-induced decrease in mRNA expression of these genes. These findings suggest that Pro-Hyp is linked to the downregulation of ubiquitin ligases associated with muscle atrophy without impacting myogenesis in response to DEX-induced muscle atrophy.

### 3.3. Pro-Hyp Attenuates Protein Levels of Muscle-Atrophy-Associated Ubiquitin Ligases and Ubiquitinated Proteins in DEX-Induced Myotube Atrophy

**Figure 3:**
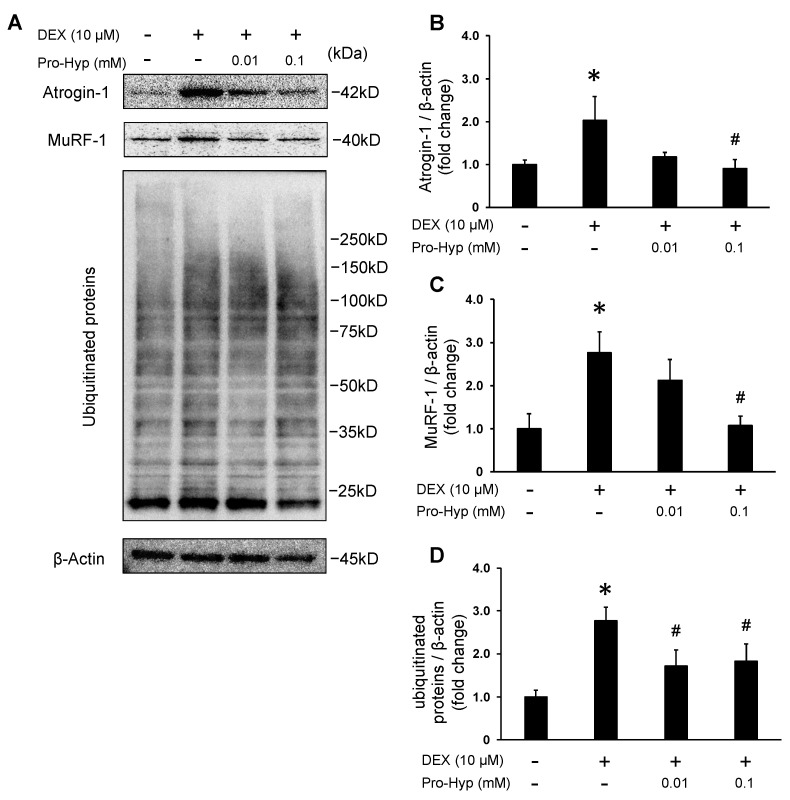
Effects of Pro-Hyp on protein levels of muscle-atrophy-associated ubiquitin ligases and ubiquitinated proteins in DEX-induced myotube atrophy. C2C12 myotubes were treated with 10 µM of DEX and Pro-Hyp (0.01 mM and 0.1 mM) for 24 h. Western blot assay examined atrogin-1, MuRF-1, and ubiquitinated protein level. β-actin was used as an internal control. (**A**) Representative Western blot of total forms of atrogin-1, MuRF-1, ubiquitinated proteins, and β-actin. (**B**) The ratio of total atrogin-1 and β-actin normalized to the control. (**C**) The ratio of total MuRF-1 and β-actin normalized to the control. (**D**) The ratio of ubiquitinated proteins and β-actin normalized to the control. Data are expressed as means ± SD. * *p* < 0.05 vs. non-treated controls, # *p* < 0.05 vs. DEX-treated groups. *p* < 0.05.

To determine whether Pro-Hyp exerts an inhibitory effect on atrophy in glucocorticoid-induced atrophic conditions, the protein levels of atrogin1 and MuRF-1 were analyzed with a Western blot. DEX treatment increased the protein levels of atrogin-1 and MuRF-1, and they were significantly higher than the control group. In contrast, the DEX + 0.01 mM Pro-Hyp treated group resulted in a 42% reduction in atrogin-1 protein expression compared to the DEX group. The DEX + 0.1 mM Pro-Hyp treated group significantly reduced the protein expression of atrogin-1 compared to the DEX-treated C2C12 myotubes (Figure 3A,B). In addition, the DEX + 0.01 mM Pro-Hyp treated group resulted in a 24% reduction in MuRF-1 protein expression compared to the DEX group. The DEX + 0.1 mM Pro-Hyp treated group significantly reduced the protein expression of MuRF-1 compared to the DEX-treated C2C12 myotubes (Figure 3A,C). To further investigate the inhibition of protein degradation with Pro-Hyp, ubiquitination was analyzed using a Western blot. The results showed that DEX treatment significantly increased the levels of ubiquitinated proteins, while co-treatment with Pro-Hyp and DEX significantly decreased ubiquitinated proteins compared to DEX treatment alone (Figure 3A,D). These results indicate that Pro-Hyp suppresses the expression of ubiquitin ligases atrogin-1 and MuRF-1, consequently inhibiting protein degradation and mitigating DEX-induced muscle atrophy.

### 3.4. Pro-Hyp Prevented DEX-Induced Muscle Atrophy through Akt/mTOR/Foxo3a Signaling

**Figure 4:**
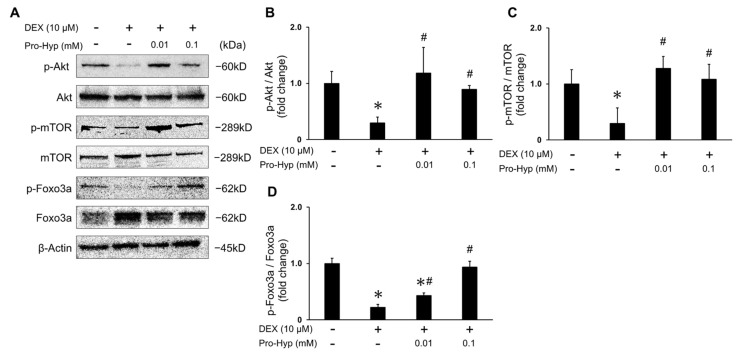
Effects of Pro-Hyp on Akt-mTOR-Foxo3a signaling in DEX-induced myotube atrophy. C2C12 myotubes were treated with 10 μM of DEX and Pro-Hyp (0.01 mM and 0.1 mM) for 24 h. Western blot assay examined Akt, phosphorylated Akt, mTOR, phosphorylated mTOR, Foxo3a, and phosphorylated Foxo3a protein levels. β-actin was used as an internal control. (**A**) Representative Western blot of phosphorylated and total forms of Akt, phosphorylated and total forms of Foxo3a, phosphorylated and total forms of mTOR, and β-actin. (**B**) The ratio of phosphorylated and total Akt normalized to the control. (**C**) The ratio of phosphorylated and total mTOR normalized to the control. (**D**) The ratio of phosphorylated and total Foxo3a normalized to the control. Data are expressed as means ± SD. * *p* < 0.05 vs. non-treated controls, # *p* < 0.05 vs. DEX-treated groups. *p* < 0.05.

To explore the mechanism of action of Pro-Hyp in preventing muscle atrophy, we analyzed the Akt, mTOR, and Foxo3a proteins, which are signaling pathway factors associated with protein synthesis and degradation, using a Western blot analysis. DEX treatment resulted in the inhibition of Akt and mTOR phosphorylation. Conversely, both the DEX + 0.01 mM Pro-Hyp and DEX + 0.1 mM Pro-Hyp treatment groups exhibited a significant increase in Akt and mTOR phosphorylation levels compared to C2C12 myotubes treated with DEX alone (Figure 4A–C). Furthermore, DEX treatment led to a significant reduction in the phosphorylation level of Foxo3a when compared to the control group. In contrast, the DEX + 0.01 mM Pro-Hyp treated group showed a significant increase in the phosphorylation level of Foxo3a compared to the DEX-treated group, although it remained significantly lower than the control group. The DEX + 0.1 mM Pro-Hyp treated group displayed a significant increase in the phosphorylation level of Foxo3a compared to the DEX-treated group, with no significant difference when compared to the control group (Figure 4A,D). These results suggest that Pro-Hyp diminishes DEX-induced muscle atrophy by modulating the Akt/mTOR/Foxo3a signaling pathway triggered by DEX.

## 4. Discussion

Muscle atrophy not only occurs in various physiological and pathological conditions, such as inactivity, muscle wasting, fasting, sepsis, cachexia, cancer, diabetes, and many chronic diseases, but it also causes increased morbidity and mortality. Therefore, maintaining healthy muscle mass is necessary for a healthy life. Recently, the possible effects of collagen peptide intake on muscle hypertrophy and prevention of muscle atrophy have been reported [25,27]. Still, there are no reports on the impact of specific peptides contained in collagen peptides on muscle atrophy or their molecular mechanisms. In this study, we report the protective activity of Pro-Hyp, a collagen-derived peptide, against DEX-induced C2C12 myotubular atrophy and its mechanism of action.

Many previous studies have shown that DEX reduces the diameter and MHC expression of C2C12 myotubes, which are known to be representative phenotypic modifiers of muscle atrophy [31,32]. In this study, we analyzed the effect of Pro-Hyp on DEX-induced C2C12 myotube atrophy by immunostaining with MHC, a representative marker of myotube differentiation [33]. As shown in Figure 1, the diameter of C2C12 myotubes decreased after DEX treatment but was restored with Pro-Hyp addition (0.01 and 0.1 mM). Thus, Pro-Hyp can prevent muscle atrophy by effectively restoring DEX-induced inhibition of C2C12 myotube differentiation.

Atrogin-1 and MuRF-1 stand out as the two most recognized muscle-specific E3 ubiquitin ligases and serve as crucial markers for muscle atrophy [5,34]. Atrogin-1 becomes evident early in the process, even before the onset of muscle weakness, and plays a role in the degradation of eukaryotic translation initiation factor 3 (eIF3), a key factor in protein translation initiation [35]. On the other hand, MuRF-1 is responsible for degrading MHC protein in DEX-treated skeletal muscle [36]. Several studies have demonstrated that DEX induces muscle atrophy in C2C12 myotubes by increasing the expression of atrogin-1 and MuRF-1 [7,37]. Conversely, myogenic factors like MyoD and Myogenin play a role in activating skeletal muscle formation and differentiation [38]. It has been reported that myoD and Myogenin expression is upregulated during myotube formation [39]. Moreover, DEX administration has been shown to decrease the expression of myoD and Myogenin and hinder myotube formation [7]. In the present study, the mRNA expression of atrogin-1 and MuRF-1 was significantly higher than in the control group. However, when Pro-Hyp and DEX were combined, the mRNA expression of atrogin-1 and MuRF-1 was also reduced (Figure 2A,B). This suppression of the DEX-induced upregulation of atrogin-1 and MuRF-1 mRNA expression with Pro-Hyp was further confirmed at the protein expression level for each respective gene (Figure 3A–C). Pro-Hyp mitigates the DEX-induced upregulation of ubiquitin ligase expression, in line with the observation that the ubiquitinated proteins, previously heightened with DEX administration, were decreased with the addition of Pro-Hyp (Figure 3A,D). Conversely, the mRNA expression levels of MyoD and Myogenin were diminished with DEX treatment compared to the control. However, the addition of Pro-Hyp did not affect the mRNA expression levels of MyoD and Myogenin, which were elevated with DEX treatment. These results suggest that Pro-Hyp effectively suppresses the expression of ubiquitin ligases atrogin-1 and MuRF-1, thereby inhibiting protein degradation with the ubiquitin–proteasome pathway and mitigating DEX-induced muscle atrophy.

Previous reports have indicated that Akt activation elevates mTOR phosphorylation, leading to an upregulation of protein synthesis in muscle tissue [40]. It has also been reported that the inhibition of the Akt/mTOR signaling pathway with DEX results in muscle atrophy [7]. Furthermore, several studies reported that collagen-derived peptides can promote muscle protein synthesis mainly through the Akt/mTOR pathway [24,29]. In the present study, DEX treatment substantially reduced the phosphorylation of Akt and mTOR compared to the control group. However, Pro-Hyp treatment significantly improved the relative phosphorylation levels of Akt and mTOR compared to the DEX-treated group (Figure 4A–C). Consequently, these results indicate that Pro-Hyp promotes the anabolic processes of muscle-specific proteins.

Prior research has indicated the involvement of the Foxo transcription factor in the regulation of atrogin-1 and MuRF-1 expression [41]. In the context of DEX-induced myotube atrophy, there is a reduction in the phosphorylation of Foxo1 and Foxo3a, leading to their translocation to the nucleus, where an upregulation occurs, subsequently increasing the expression of atrogin-1 and MuRF-1. Another relevant factor is the kinase Akt, which has been shown to phosphorylate Foxo during myotube atrophy. This phosphorylation hinders the translocation of Foxo to the nucleus, thereby preventing the upregulation of atrogin-1 and MuRF-1 [42]. Our previous research has identified a mechanism through which Pro-Hyp facilitates the interaction between Foxo1 and Runx2, a master regulator of osteoblast differentiation. This interaction leads to the stimulation of Runx2 promoter activity, ultimately renewing osteoblast differentiation [43]. Notably, Kitakaze et al. demonstrated that Hyp-Gly, a dipeptide derived from collagen, triggers myogenic differentiation by activating the Akt signaling pathway [24]. These reports suggest a potential involvement of collagen-derived dipeptides in intracellular Akt/Foxo signaling. In the present study, the administration of Pro-Hyp prevented the DEX-induced decreases in Akt phosphorylation and Foxo3a phosphorylation (Figure 4A–C). These findings indicate that Pro-Hyp administration inhibits DEX-induced muscle atrophy in C2C12 cells by impeding ubiquitin E3 ligases through the Akt-Foxo3a signaling pathway.

Furthermore, a recent study documented the suppression of DEX-induced muscle atrophy in mice through CP supplementation [28]. In the study, CP effectively curbed muscle atrophy while not impacting the expression of ubiquitin ligases involved in muscle degradation. While these findings contrast with our present study, some investigations employing CP and collagen tripeptide (CTP), which contains elevated collagen tripeptide levels, have indicated that the administration of CP and CTP was regulated in different ways to ameliorate muscle loss caused by aging [29]. These outcomes propose that collagen hydrolysates, comprising peptides of diverse molecular sizes and specific sequences, might offer various mechanisms to counteract muscle atrophy. Subsequent research should also investigate the effects of different molecular sizes and other collagen-derived oligopeptides in inhibiting muscle atrophy. Moreover, several human studies have consistently demonstrated that CP, combined with resistance exercise, leads to amplified muscle strength and diminished muscle weakness [25,27]. It would be beneficial for future research to investigate whether the administration of Pro-Hyp, in combination with resistance exercise, proves effective in mitigating muscle atrophy.

While the current findings support the use of DEX-treated C2C12 myotubes as an in vitro model of muscle atrophy, it is important to acknowledge several potential limitations in this study. First, this study solely investigated and exclusively focused on C2C12 myotubes. We suggest that conducting additional research involving other cell lines, such as L6 myotubes (a rat cell line), or using experimental animals would provide a more comprehensive evaluation of the impact of Pro-Hyp on glucocorticoid-induced muscle wasting and the regulation of myoprotein degradation. Another limitation of the study is its exclusive focus on the ubiquitin–proteasome system as the mechanism of proteolysis. We did not have the opportunity to investigate the impact of Pro-Hyp on muscle proteolysis mediated with catabolic pathways such as the autophagy–lysosome system [44]. Furthermore, our examination was limited to the Akt/mTOR/Foxo3a signaling pathway as the mechanism through which glucocorticoids induce atrophy. It is worth noting that glucocorticoids have been implicated in contributing to muscle atrophy by activating inflammatory signaling pathways [45], although the effects of Pro-Hyp on these pathways has not been explored.

## 5. Conclusions

In conclusion, the present study provides molecular evidence that Pro-Hyp improves DEX-induced atrophy in C2C12 myotubes through the regulation of the Akt/mTOR/Foxo3a signaling pathway, resulting in the inhibition of the upregulation of the ubiquitin ligases atrogin-1 and MuRF-1. However, further studies are required to investigate other possible anti-atrophy-related mechanisms and in vivo efficacies for Pro-Hyp. Overall, this study highlights the potential importance of Pro-Hyp as an effective therapeutic target for DEX-induced muscle atrophy by decreasing muscle-specific ubiquitin ligase expression.

## Data Availability

All relevant data are within the paper and Supplementary Materials.

## Supplementary Materials

The following supporting information can be downloaded at: https://www.mdpi.com/article/10.3390/biom13111617/s1.
