# Supplementary material for: Preventive Effects of Collagen-Derived Dipeptide Prolyl-Hydroxyproline against Dexamethasone-Induced Muscle Atrophy in Mouse C2C12 Skeletal Myotubes
Source: Biomolecules. 2023 Nov 5;13(11):1617. doi: 10.3390/biom13111617 (PMC10669392; doi:10.3390/biom13111617)

Figure 3A atrogin-1

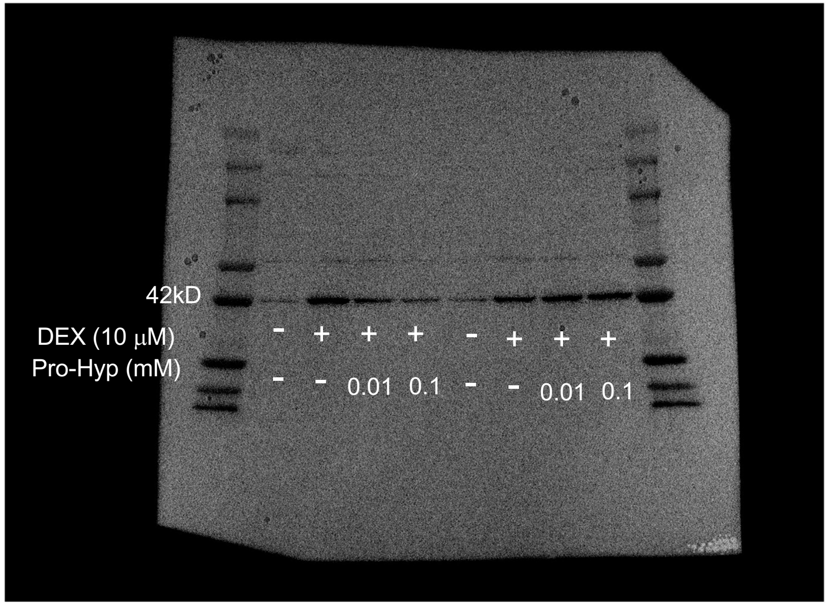

Figure 3A atrogin-1

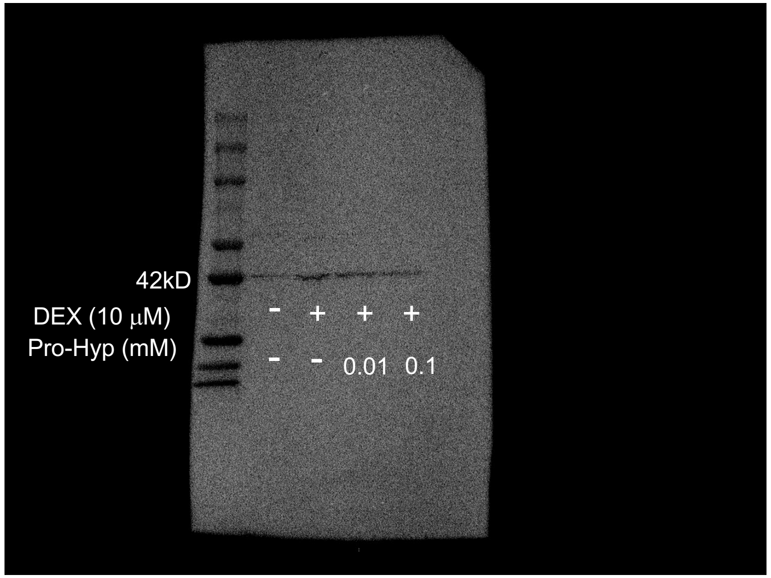

Figure 3A MuRF-1

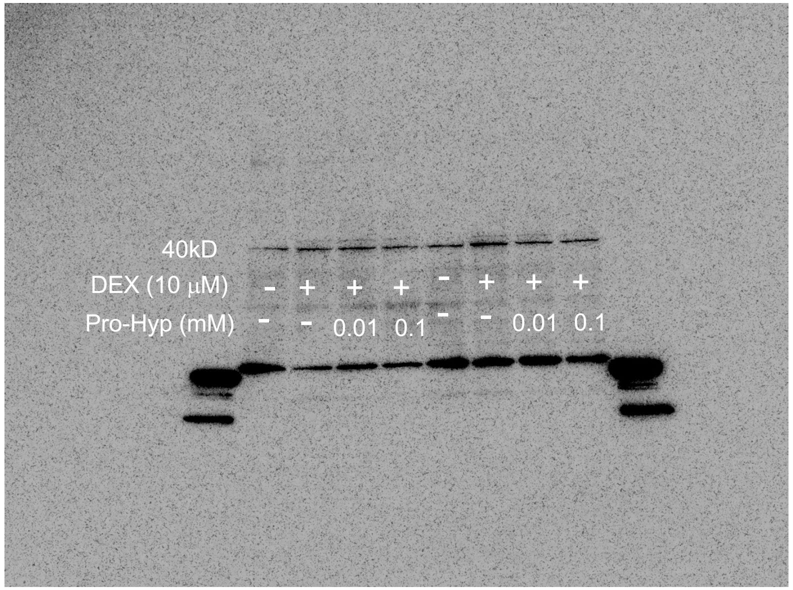

Figure 3A MuRF-1

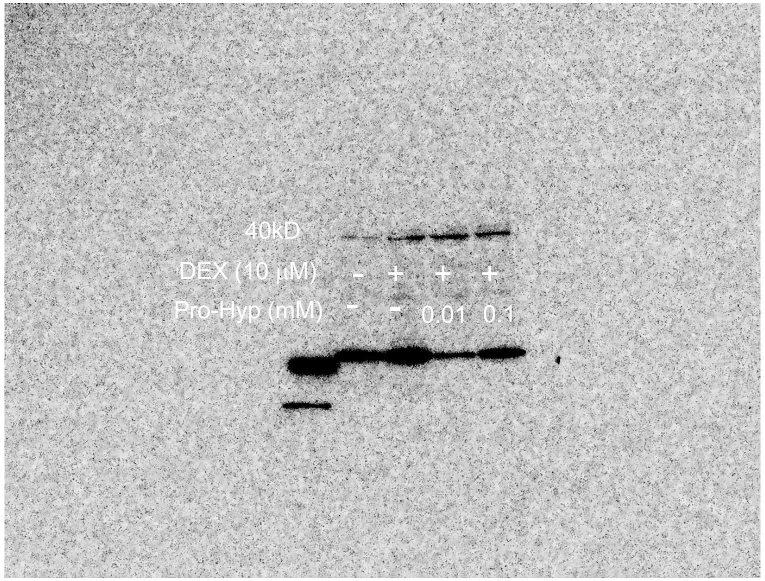

Figure 3A Ubiquitinated proteins

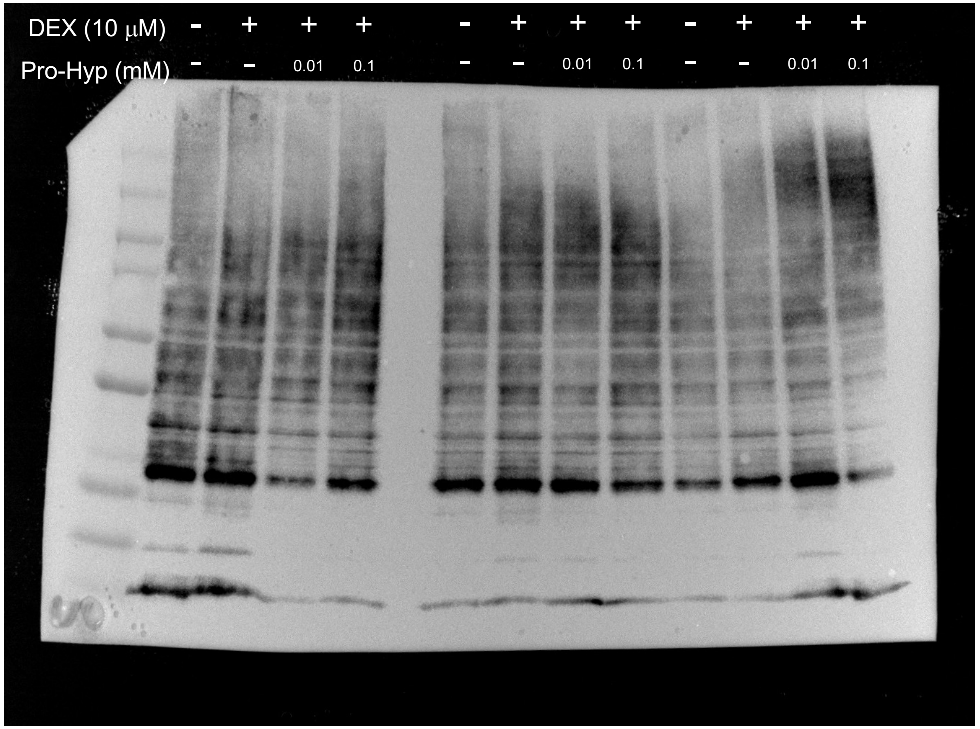

Figure 3A 4A β-actin

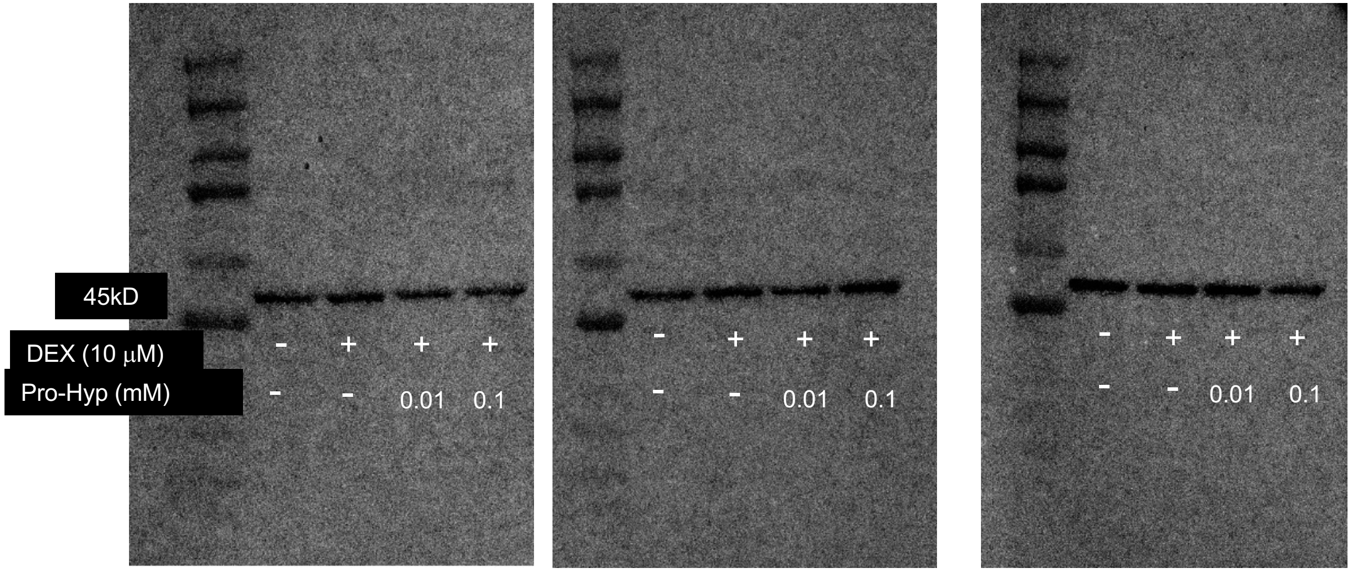

Figure 4A p-Akt

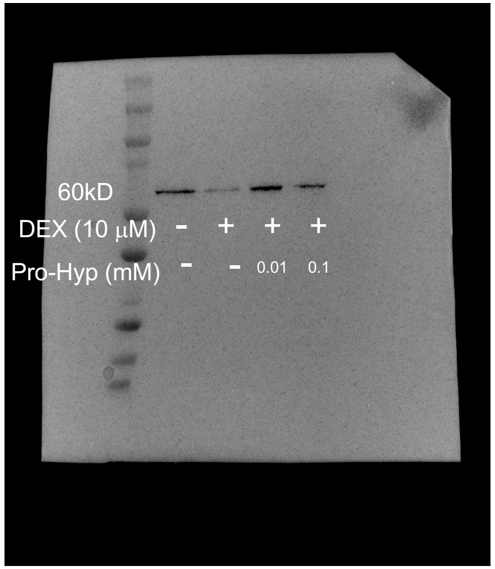

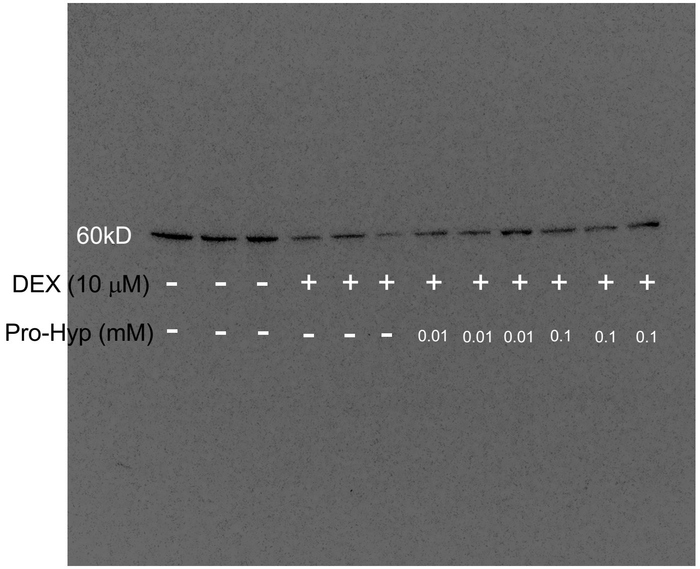

Figure 4A Akt

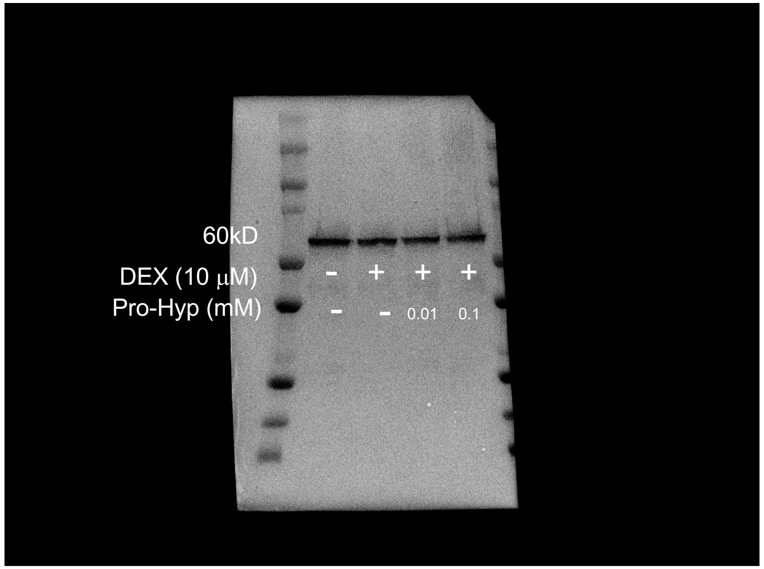

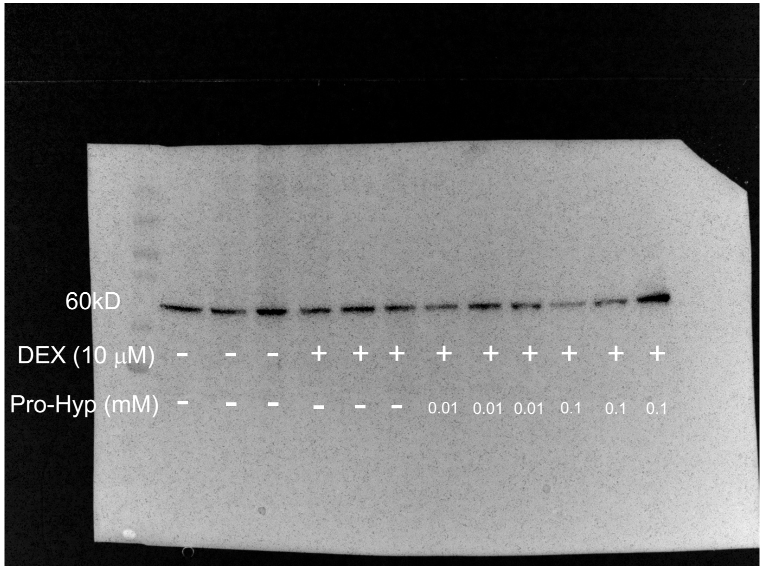

Figure 4A p-mTOR

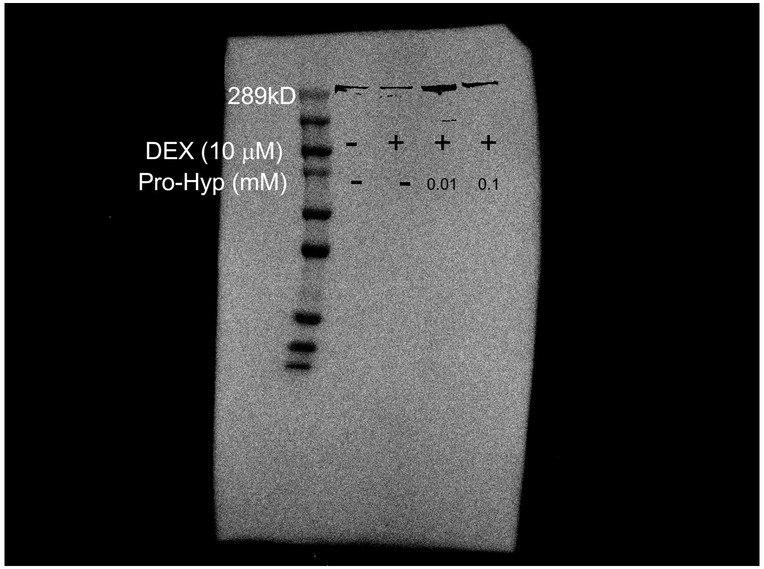

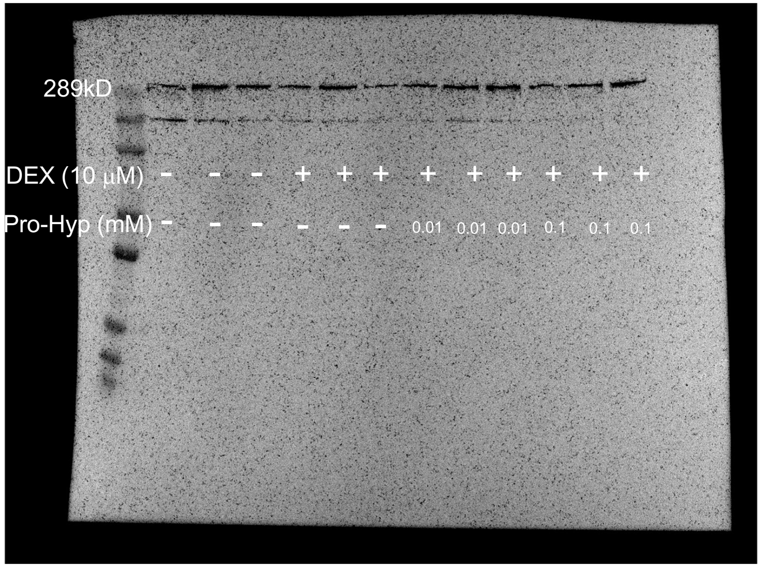

Figure 4A mTOR

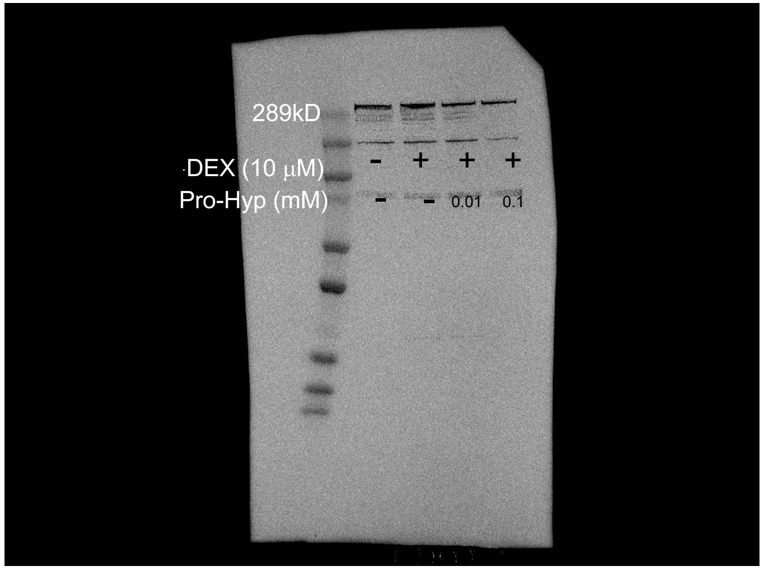

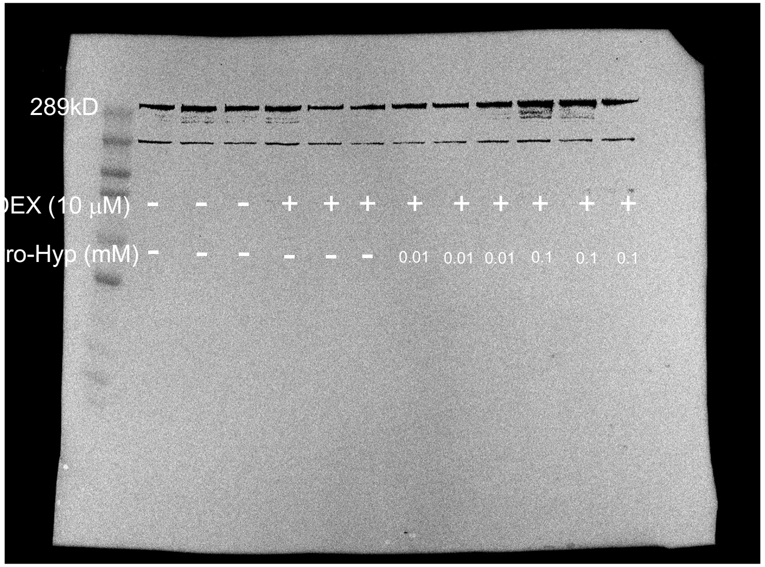

Figure 4A p-Foxo3a

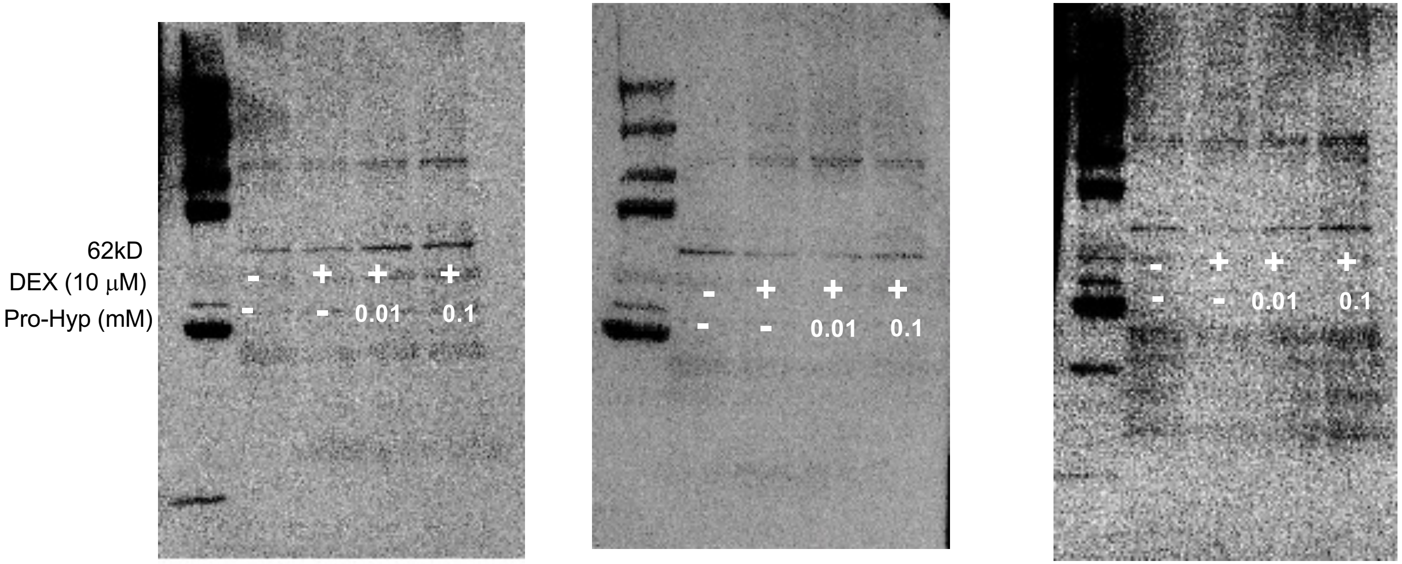

Figure 4A Foxo3a

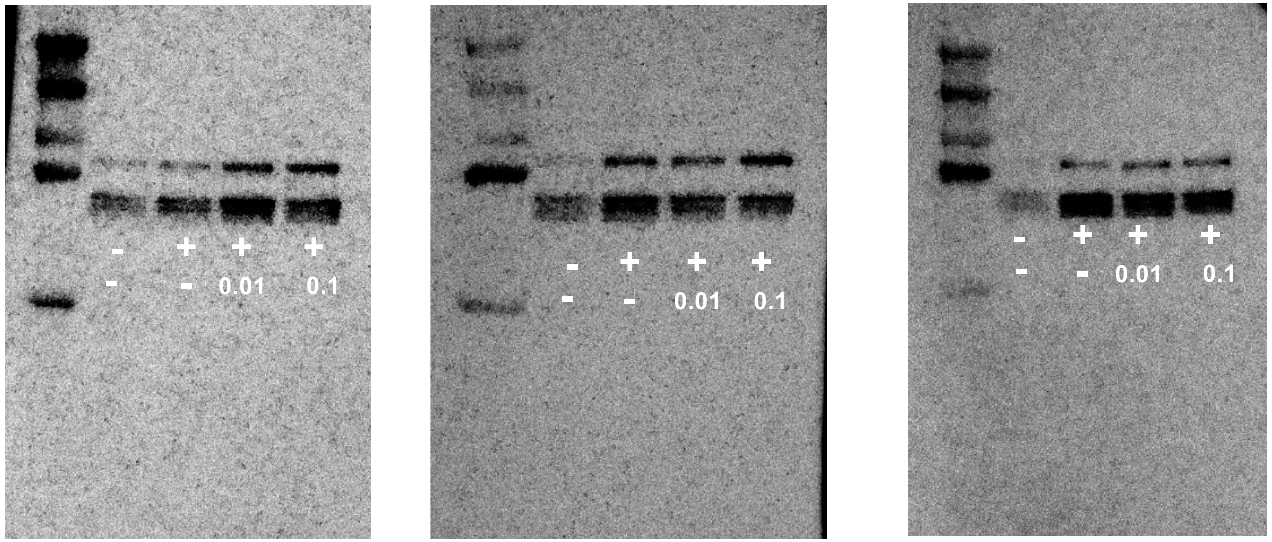

Supplement: Supplementary file 1 [file biomolecules-13-01617-s001.zip › biomolecules-2590267-supplementary.pdf]
